# Genetic diversity and drug susceptibility of *Mycobacterium tuberculosis* in a city with a high prevalence of drug resistant tuberculosis from Southeast of Mexico

**DOI:** 10.1186/s12879-021-06904-z

**Published:** 2021-11-30

**Authors:** Roberto Zenteno-Cuevas, Daniela Munro-Rojas, Damián Pérez-Martínez, Esdras Fernandez-Morales, Ana C. Jimenez-Ruano, Hilda Montero, Leila Escobar, Everest de Igartua, Ángel Trigos, Javier Fuentes-Dominguez

**Affiliations:** 1 Public Health Institute, University of Veracruz, Av. Luis Castelazo Ayala S/N, A.P. 57, Col. Industrial Ánimas, Xalapa, 91190 Veracruz, México; 2 Multidisciplinary Research Network on Tuberculosis, Veracruz, Mexico; 3 Doctorate in Health Sciences Program, Health Sciences Institute, University of Veracruz, Veracruz, Mexico; 4 Anáhuac University, Xalapa, Veracruz, Mexico; 5 Master of Health Science Program, Health Sciences Institute, University of Veracruz, Xalapa, Veracruz, Mexico; 6 Veracruz Health Department, Veracruz, Mexico; 7 Research Center in Applied Mycology, University of Veracruz, Xalapa, Veracruz, Mexico

**Keywords:** Spoligotyping, MIRU-VNTR, Genotyping, Tuberculosis, Mexico

## Abstract

**Background:**

Mexico is on the top five countries with the highest number of TB cases in America continent, nevertheless, information about genotypes circulating is practically unknown. Considering the above this study aims to characterize the genetic diversity of TB in the city of Veracruz, México.

**Methods:**

A cross-sectional study was conducted among positive smear samples from patients living in Veracruz City, samples were cultured, and first-line drug profiles determined. Genotyping was made by spoligotyping and MIRU-VNTR 24 loci. Associations of lineages, clusters, and variables were also analyzed.

**Results:**

Among the 202 isolates analyzed resistance to at least one drug was observed in 60 (30%) isolates and 41(20%) were multidrug-resistant. Three major lineages were identified: L4/Euro-American (88%), L1/Indo-Oceanic (9%), and L2/East Asian (3%). The Euro-American lineage included more than six sublineages, the most abundant were: H (32%), T (23%), LAM (18%), and X (12%). 140 isolates (70%) were placed in 42 SITs patterns.

**Conclusions:**

These results provide the first baseline data on the genetic structure of TB in the city of Veracruz. Sublineages H, X and LAM were predominant; however, it was founded an important diversity of genotypes that could contribute to the dispersion of TB and explain the high prevalence. This information might be useful for the development of further interventions to reduce impact of TB.

**Supplementary Information:**

The online version contains supplementary material available at 10.1186/s12879-021-06904-z.

## Background

The World Health Organization (WHO) report describes that tuberculosis was responsible for more than 10 million cases and 1.4 million deaths in 2019, this data highlight the impact of this infectious disease [1]. In this context, in 2018 Mexico had more than 29,000 new cases of TB with an incidence of 23 per 100,000 inhabitants. The multidrug resistant/rifampicin resistant (MDR-TB /RRTB) number of cases was 950 with an incidence of 0.75 per 100,000 inhabitants, the proportion of new cases with MDR/RR was 2.6%, that increases to 11% in previously treated cases [1]. These figures place Mexico in the top five countries with the highest numbers of TB and MDR-TB in Latin America.

According to the official data, in 2018 Veracruz state presented near to 2400 cases of pulmonary tuberculosis; the incidence was 29 per 100,000 inhabitants, placing the state among the highest number of tuberculosis cases in the country. Specifically, Veracruz City and its metropolitan area, annually represent about 30% of the state’s cases of pulmonary tuberculosis, reaching an incidence rate of 42.15 per 100,000 inhabitants, twice the national rate.

The use of molecular typing tools, such as MIRU-VNTR and spoligotyping, allow the genotypic characterization of TB isolates. This information is useful to understand the population structure of *M. tuberculosis* circulating within specific regions [2, 3], and identify lineages, such as Beijing, with a tendency to expand rapidly in the population or to generate drug resistance [4–7].

Information related to the genotypic characterization of tuberculosis in México is limited. Most of the reports describe the Euro-American lineage (L4) as the highest found, and to a lesser extent are lineages Indo-Oceanic (L1) and East Asian (L2), with important variations according to the state or city, reflecting the specific geographical and social characteristics of this disease [8–18]. Nevertheless, there are no information regards to the genotypic characteristics of the isolates circulating in Veracruz city, therefore the goal of this work is to describe the genetic structure of tuberculosis circulating in one of the cities with the highest TB prevalence in Mexico.

## Methods

### Population and clinical sample isolation and DNA

A total of 239 individuals living in the Veracruz City and metropolitan area, and with a confirmed acid-fast bacilli smear positive sputum specimen were included in the study. Samples were randomly recovered throughout the period April 2013 to May 2016 by the medical staff of the tuberculosis program from the Veracruz Health Department. The city of Veracruz and metropolitan area have a population close to 600,000 inhabitants.

Sputum samples were decontaminated using Petroff´s modified method [19] and primary isolation was made in Lowenstein-Jensen medium. Susceptibility testing for the first-line drugs streptomycin (S), isoniazid (H), rifampin (R), ethambutol (E), and pyrazinamide (Z) was performed using the fluorometric method (MGIT 960, Becton–Dickinson).

Variables such as age, gender, place of residence, and treatment, were recovered from the respective clinical summary of the patients. No physical interventions took place with the patients, and all information collected was anonymized and treated as confidential.

### Isolation and purification of *M. tuberculosis* genomic DNA

DNA isolation was done with a loop of cultured mycobacteria, following the recommendations of Van Soolingen et al*.* [20]. The purified DNA was dissolved in TE buffer (10 mM Tris–HCl, 1 mM EDTA, pH = 8.0) and quantified with the Nanodrop™ 2000 Spectrophotometer (Thermo Fisher Scientific, Waltham, USA). The DNA samples were diluted to the required concentration and stored at -20 °C until use.

### Spoligotyping

Spoligotyping was carried out by duplicated following standard techniques [21] using the Spoligotyping Kit (Ocimum Biosolutions, Hyderabad, India). The DNA from *M. tuberculosis* H37Rv and *M. bovis* BCG were used as controls.

Spoligotype international type (SIT) and phylogenetic clades (sublineages and families) were assigned according to the SITVIT2 http://www.pasteur-guadeloupe.fr:8081/SITVIT2/ [22] and the similarity search module of MIRU-VNTRplus platform [23, 24]. In those isolates with not definition of SIT and lineage, the assignment was done by conformational bayesian networks (CBN), using the online tool “TB-lineage”, according authors recommendations [25]. Final lineage and sublineage assignment was done considering recommendations by Coll et al., and Stucky et al., [26, 27]. Clustering rate was calculated using the formula (nc -c)/n, where the nc is the total number of clustered cases, c is the number of clusters, n is the total number of cases studied.

### MIRU-VNTR typing

MIRU-VNTR analysis was carried out at 24 loci, with primers and amplification conditions according to recommendations of Supply et al. [3]. For PCR set-up, reagents from the PCR Master Mix (Promega, USA) were used. The 24-loci MIRU-VNTR fragment sizes were estimated by comparison with 100 bp DNA molecular weight ladders.

Results were independently verified by two separate individuals, and the number of repeats at each locus was calculated by applying the corresponding conversion table [28]. Twenty-four-digit MIRU-VNTR codes were by last analyzed by the MIRU-VNTRplus platform [23, 24]. The discriminatory power was measured for each locus using a calculator (https://www.hpabioinfotools.org.uk/cgi-bin/DICI/DICI.pl), based on the Hunter-Gaston Diversity Index (HGDI) [29]. According to the HGDI value, each locus was classified as highly, moderately, and poorly discriminative (> 0.6; 0.3 < H < 0.6; and < 0.3, respectively) [30].

### Dendrogram and clustering

The phylogenetic tree was built using data from spoligotyping and MIRU-VNTR typing data, considering the recommendations of the specific module in the MIRU-VNTRplus platform, using the UPGMA algorithm [23, 24]. Tree polar arrangement was finally done with iTol [31].

The spoligotype cluster was defined when two or more isolates sharing identical typing profile, considering the spoligotype pattern only, a relaxed cluster definition, considering differences in three markers was considered.

### Statistical analysis

The patients’ data were analyzed using frequencies and Chi-square statistics with Yates’s correction and Fisher’s exact test. With the intention of search for possible associations of some epidemiological variables (sex, age, drug resistance, etc.) with a specific SIT or cluster, respective odd ratios were calculated, considering a value of *p* < *0.05* to be significant. All calculations were performed using the SPSS V.12 software.

### Ethical concerns

Clinical samples were taken as part of routine diagnoses, no physical interventions took place and informed consent was obtained from all subjects, and all the information collected was treated as confidential according to national regulations and following the declaration of Helsinki. The ethics committee of the Public Health Institute at the University of Veracruz approved the ethical issues involved in this study.

## Results

### Demographic characteristics of the patients

Out of the 239 patients initially included in the study, positive culture grows was observed in 202 samples from the same number of individuals, which were further studied. According to gender 138 (68%) were male. The average age was 42 ± 15 years old. All individuals claimed to have been born in the state and live in Veracruz City or metropolitan area for the last 10 years. Unemployment was mentioned by 25 individuals (11%). Alcohol intake was reported by 17 individuals (8%) (Table 1). The most frequent comorbidity was type 2 diabetes mellitus, present in 42 individuals (21%). Eleven individuals (5%) mentioned having had a previous TB infection, and 191 (95%) were classified as a new case of TB.

**Table 1:** Socio-demographic and clinical characteristics of patients with sensible and DR-TB from Veracruz city, Mexico

Variable		Total *n* = 202 (%)	Sensible-TB *n* = 141 (%)	DR-TB *n* = 61 (%)	Fisher exact test	*p*	P*
Sex	Male	139 (68)	93 (65)	46 (75)	0.246	0.184	0.245
	Female	63 (32)	48 (34)	15 (25)			
Age (years)	Mean ± SD	42 ± 15	43 ± 16	42 ± 14	0.743	0.743	0.871
	≥ 35	139 (68)	98 (70)	41 (67)			
	< 35	63 (32)	43 (30)	20 (33)			
Alcoholism	Yes	17 (8)	14 (10)	3 (5)	0.405	0.255	0.390
	No	185 (92)	127 (90)	58 (95)			
T2DM comorbidity	Yes	42 (21)	34 (24)	8 (13)	0.128	0.089	0.131
	No	160 (79)	107 (52)	53 (87)			
HIV comorbidity	Yes	2 (1)	1 (0.5)	1 (0.5)	0.506	0.527	0.883
	No	200 (99)	140 (99.5)	60 (99.5)			

^*^Yates’s correction

### Drug resistance profiles

Sensitivity against first-line drugs was observed in 142 isolates (67%) and resistance to at least one first-line drug was detected in 60 isolates (30%). Resistance to streptomycin was observed in 36 isolates (18%), to isoniazid 52 (25%), rifampicin 41 (20%), ethambutol 19 (9%) and pyrazinamide 25 (12%). Resistance to all first-line drugs SIREP and simultaneous resistance against isoniazid and rifampicin (MDR-TB) was found in 10 (12%) and 38 strains (18%) respectively. No significant associations between some epidemiological and clinical variables and drug resistance was observed in these individuals (Table 1).

### Analysis of spoligotyping and clustering

Out of 202 isolates analyzed, 86 different spoligotypes patterns were observed and 42 patterns were related with a previous SIT. By looking lineage signature by CBN, in the isolates where was not possible to identify the respective SIT, it was possible to assign the sublineage in almost all isolates analyzed (Table 2).

**Table 2:** Spoligotype, SITs and lineages of *Mycobacterium tuberculosis* isolates from Veracruz City, Mexico

SIT	Lineage / Sublineage	No isolates (%)	No. DR/ MDR	Octal code	Global Distribution	Mexico Distribution**	Refs
	*Indo-Oceanic (L1.2)*						
19	EAI2-Manila	10 (5%)	4/1	677,777,477,413,771	SGP. JAM, SAU, SWE, TWN. TUN, USA, JPN, GBR, PHL, ITA, AUT, THA, BEL, CZE, ARG, FRA, MEX,	NUE, GUE, SAN, JAL, EDO, VER	[8, 9, 15, 17]
3277	EAI2-Manila	1 (0.5)	–	777,777,477,513,771	MEX	VER	[14]
–	EAI2-Manila	2 (1)		677,177,477,403,771	–	VER	–
–	EAI2-Manila*	2 (1)	1/1	037,777,477,413,771	–	VER	–
–	EAI2-Manila*	1 (0.5)	–	637,057,476,013,061	–	VER	–
–	EAI2-Manila*	1 (0.5)	–	777,777,477,417,771	–	VER	–
–	EAI3-IND*	1 (0.5)	–	437,776,774,010,000	–	VER	–
	*East Asian (L2.2)*						
1	Beijing	3 (1.4)	1/1	000,000,000,003,771	FRA, POL, ARG, CUB, USA, BRA, VEN, KAZ, RUS, MEX	NUE, SAN, EDO, BAJ, VER, JAL	[10, 17]
–	MANU2*	1(0.5)	1/1	102,276,416,563,771	–	VER	–
–	MANU3*	1 (0.5)	–	702,376,717,770,771	–	VER	–
–	MANU3*	1 (0.5)	1/1	776,377,677,770,771	–	VER	–
	*Euro American, X (L4.1.1- 4.1.1.3)*						
92	X3	3 (1.4)	-	700,076,777,760,771	BRA, MEX, DNK, ARG, HND, ZAF,TWN, NLD, TUN, AUS, USA, BRA, CUB, NZL, ZWE, IND, NLD, PER, JPN, PRY, ITA, ESP, ESP, AUT, MOZ,GNB, RUS	NUE, GUE, JAL, TAM, VER, BAJ	[8, 10, 12, 15–17]
119	X1	8 (4)	3/3	777,776,777,760,771	BRA, FRA, MEX, JAM, SUR, TTO, SAU, ALB, ZAF, DOM, TUN, USA, GRC, IND, BRA, NLD, CAN, LVA, COL, ITA	NUE, JAL, EDO, TAM, YUC, SIN, VER	[8, 10, 11, 15]
3278	X3	4 (2.5)	4/4	700,076,717,760,771	ESP, MEX	VER*	[17]
1756	X3	1 (0.5)	–	700,022,777,760,771	PRT, USA	VER	–
–	X1	1 (0.5)	–	777,176,777,760,771	–	VER	–
–	X1*	1 (0.5)	–	177,576,777,760,771	–	VER	–
–	X1*	1 (0.5)	–	753,014,777,760,331	–	VER	–
–	X2*	2 (1)	–	777,356,376,020,601	–	VER	–
–	X3*	1 (0.5)	–	700,014,774,020,771	–	VER	–
–	X3*	1 (0.5)	–	400,037,774,060,771	–	VER	–
–	X3*	1 (0.5)	1/1	700,076,703,760,771	–	VER	–
–	X3*	1 (0.5)	–	702,077,717,770,770	–	VER	–
	*Euro American, Haarlem (4.1.2)*						
2	H2	7 (3.4)	1/0	000,000,001,020,771	USA, FRA, BRA, NLD, ITA, ZAF, IND, SWE, TUR, GMB, MEX	NUE, GUE, SAN, JAL,VER	[9, 12, 15–17]
3	H3	5 (1.6)	–	000,000,007,729,771	FRA, MEX, ETH, ARG, USA, CUB, COL, BRA, ITA, VEN	NUE, BAJ, JAL, VER	[8, 17]
46	H/U	3 (1.4)	–	777,777,770,000,000	DNK, SAU, POL, ARG, DEU, BRA, PAN, PAN, IDN, MEX, BRA, FRA, PER, TUR, ITA, ESP, CZE, ZAF, FIN, ITA, RUS	NUE, GUE, EDO, VER	[8, 9]
47	H1	1 (0.5)	–	777,777,774,020,771	BRA, FRA, MEX, DEU, BEL, MAR, PRT, JAM, SUR, POL, BGR, RUS, ETH, ARG, NLD, TUN, AUT, ITA, USA, CUB, SWE, ESP, GNB, NLD	NUE, GUE, JAL, GUA, BAJ, SIN, VER	[9, 16, 17]
50	H3	18 (9)	6/3	777,777,777,720,771	FRA, MEX, DEU, BEL, POL, RUS, ITA, DNK, TUR, BRA, ARG, DOM, AUT, USA, CUB, ESP, JPN, COL, PAN	NUE, GUE, SAN, JAL, EDO, BAJ, COL, PUE, VER	[8, 9, 15–17]
602	H1	1 (0.5)	1/1	777,777,770,000,771	BRA, BEL, MAR, POL, RUS, TUR, DEU, USA, ESP, NZL, GEO, USA, ALB	VER*	[17]
450	H1	1 (0.5)	–	777,776,770,000,000	CMR, SWE, USA, MEX, PER, ITA, PRT, JAM, SUR, TTO, POL, KAZ,	NUE, BAJ, CDM, VER	[8, 17]
472	H3	1 (0.5)	–	377,777,777,720,771	BRA, BEL, CZE, USA, ESP, NLD, FRA	–	–
571	H1	1 (0.5)	–	777,775,774,020,771	USA	–	–
602	H1	1 (0.5)	–	777,777,770,000,771	BEL,POL,BGR,MAR,SAU,NLD,USA, TUR,NZL,BRA,IRN,ITA,ZAF,GEO	–	–
849	H3	1 (0.5)	–	637,777,777,720,771	SEN, DEU, CAMR, USA, PER, GMB, ITA	–	–
948	H3	1 (0.5)	1/1	777,777,760,020,611	USA, MEX, ITA, ESP, PER	–	–
1539	H3	2 (1)	1/1	773,777,777,720,771	MAR, ESP, MYS, MOZ	CDMX	–
–	H3*	1 (0.5)	1/1	777,350,376,020,671	–	VER	–
–	H1*	2 (1)	–	776,067,770,000,731	–	VER	–
–	H1*	2 (1)	–	776,377,760,000,731	–	VER	–
–	H1*	3 (1.4)	–	777,156,340,020,601	–	VER	–
–	H1*	3 (1.4)	–	777,377,770,000,000	–	VER	–
–	H1*	2 (1)	–	777,637,774,120,731	–	VER	–
–	H2*	2 (1)	–	000,002,004,020,771	–	VER	–
–	H3*	1 (0.5)	–	772,777,774,500,600	–	VER	–
–	H3*	2 (1)	–	777,677,777,720,671	–	VER	–
	*Euro-American, TUR (4.2.2)*						
41	Turkey	2 (1)	1/1	777,777,404,720,571	DEU,BEL,DNL,TUR,SAU,ETH,SWE,ROU,NLD, ITA, SAF,BGR	–	–
	*Euro-American, LAM (4.3)*						
33	LAM3	4 (2)	2/2	776,177,607,760,771	BRA, FRA, BEL, ZAF, MAR, PRT, VEN, ITA, SAU, ARG, HND, ITA.USA, CUB.ESP, NLD, COL, PAN, NLD, PER, GMB, MOZ	NUE, GUE, SAN, JAL, SIN, VER	[8–10, 12, 17]
42	LAM9	15 (7)	8/5	777,777,607,760,771	BRA, IND, FRA, MEX, BEL,, VEN, RUS, POL, ARG, HND, SWE, ITA, USA, CUB, TUR, ESP, COL, PAN, HTI, GNB, ZWE, TUR, BEL	NUE, GUE, JAL, EDO, BAJ, TAM, SIN, GUA, CHI, VER	[8, 9, 11, 12, 15, 16]
130	LAM	1 (0.5)	–	776,177,607,760,731	BRA,MEX,ZAF,HND,PER,ESP,USA,COL,VEN,ITA	–	–
376	LAM3	2 (1)	1/1	376,177,607,760,771	BEL, JAM.HND, USA, ESP, BRA, ZAF, VEN	–	–
398	LAM4	1 (0.5)	1/1	777,777,607,760,631	BRA, CUB.USA, MEX, AUT, BEL, FRA	NUE, GUE, GUA, VER	[9, 17]
1076	LAM1	1 (0.5)	–	007,777,607,760,771	RUS, MOZ, BRA, IDN	–	–
1535	LAM9	1 (0.5)	–	777,577,607,760,771	BRA, BEL, ARG, ESP, USA, FRA	–	–
1718	LAM1	1 (0.5)	–	677,776,607,760,771	BRA, VEN	–	–
2350	LAM3	4 (2)	2/1	700,017,607,760,771	USA	–	–
–	LAM11-ZWE*	2 (1)	1/1	677,037,606,000,031	–	VER	–
–	LAM*	1 (0.5)	–	731,777,607,760,771	–	VER	–
–	LAM1*	1 (0.5)	–	037,777,607,760,770	–	VER	–
–	LAM3 *	1 (0.5)	–	674,037,607,760,771	–	VER	–
–	LAM3*	1 (0.5)	–	770,017,607,760,771	–	VER	–
	*Euro-American, S (L4.4)*						
707	S	2 (1)	–	736,377,777,760,771	ARG, NLD, ESP, IDN	VER*	[17]
–	S*	1 (0.5)	–	666,017,777,760,771	–	VER	–
–	S*	1 (0.5)	–	770,077,770,700,731	–	VER	–
	*Euro-American, T (L4.7-4.8)*						
37	T3	3 (1)	–	777,737,777,760,771	FRA, DEU, BEL,, VEN, POL, RUS, LBY, DNK, SAU, ETH, BRA, SWE, SWE, ESP, USA, TUR, COL, GBR, YEM, IND, NLD, ITA, ZAF, BRA, JPN, DEU, CZE, AUT, MOZ, ZAF, BEL, CHN, ETH, ITA, IRQ	SAN, VER	[12, 17]
51	T1	1 (0.5)	1/0	777,777,777,760,700	BRA, VEN, ITA, ARG, ESP, USA, GLP, FRA, PER, JPN, AUT, CHIN, IRN	GUE, JAL, EDO, VER	?
53	T1	17 (8)	2/2	777,777,777,760,771	BRA, FRA, MEX*,* DEU, BEL, MAR, GUY, JAM, POL, RUS, ITA, LBY, DNK, ARG, HND, ZAF, SWE, DOM, TZA.TUN, PER, USA, IRN, CUB, TUR, ESP	NUE, GUE, SAN, JAL, EDO, BAJ, OAX, SIN, GUA, VER	[8, 9, 12, 15]
58	T2	1 (0.5)	1/1	000,000,177,760,771	MEX, USA	BAJ	[13]
239	T2	3 (2)	1/1	777,777,777,760,031	MEX, BRA, VEN, USA, VEN, PER, BGD,	NUE, SAN, GUE, VER	[8, 9, 12, 17]
244	T1	7 (3.4)	1/0	777,777,777,760,601	BRA, IND, BEL, PRT, HTI, BGD, GNB, BRA, NLD, ZAF, FRA, GNB, ITA	VER*	[17]
334	T1	1 (0.5)	–	577,777,777,760,771	IND, BEL, KAZ, SWE, TUN, SEN, CHN, GBR, GUF, ITA, ZAF, GEO, ESO, MEX*,* CHN, GNB, FRA,	VER*	[17]
2900	T1	1 (0.5)	–	777,777,763,760,771	RUS, TUR, ALB, ITA, NGA, RUS	–	–
1123	T1-RUS2*	1 (0.5)	–	770,000,777,740,171	BEL, AUS, USA,	–	–
–	T*	1 (0.5)	–	637,017,763,660,771	–	VER	–
–	T*	1 (0.5)	–	677,775,777,760,771	–	VER	–
–	T*	1 (0.5)	–	771,057,777,760,671	–	VER	–
–	T*	1 (0.5)	–	777,177,737,760,771	–	VER	–
–	T1*	1 (0.5)	1/1	770,077,777,760,771	–	VER	–
–	T1*	1 (0.5)	–	777,357,777,760,671	–	VER	–
–	T2*	1 (0.5)	–	777,777,675,760,031	–	VER	–
–	T1-RUS2 *	1 (0.5)	–	402,202,777,720,771	–	VER	–
–	T1RUS2*	1 (0.5)	1/1	710,007,747,720,771	–	VER	–
–	T1RUS2*	1 (0.5)	–	771,000,677,720,771	–	VER	–
–	T1RUS2*	1 (0.5)	1/1	771,040,377,760,771	–	VER	–
–	T3*	1 (0.5)	–	311,077,777,760,661	–	VER	–
	*Euro- American, T (L4.9)*						
451	H37Rv	1 (0.5)	–/–	777,777,477,760,771	MAR, PRT, SAU, ARG, SWE, FRA, TUN, USA, GBR, EGY, NLD, BRA, MEX, VEN, MDG, MMR, BGR,	JAL, VER	[15, 17]

*Lineage/sublineage determined by conformational bayesian network. **COL: Colima. BAJ: Baja California. CDMX: Ciudad de México. EDO: Estado de México. GUE: Guerrero. JAL: Jalisco. NUE: Nuevo León. PUE: Puebla. SAN: San Luis Potosí. SIN: Sinaloa. TAM: Tamaulipas. VER: Veracruz. YUC: BY Yucatán

**Fig. 1:**
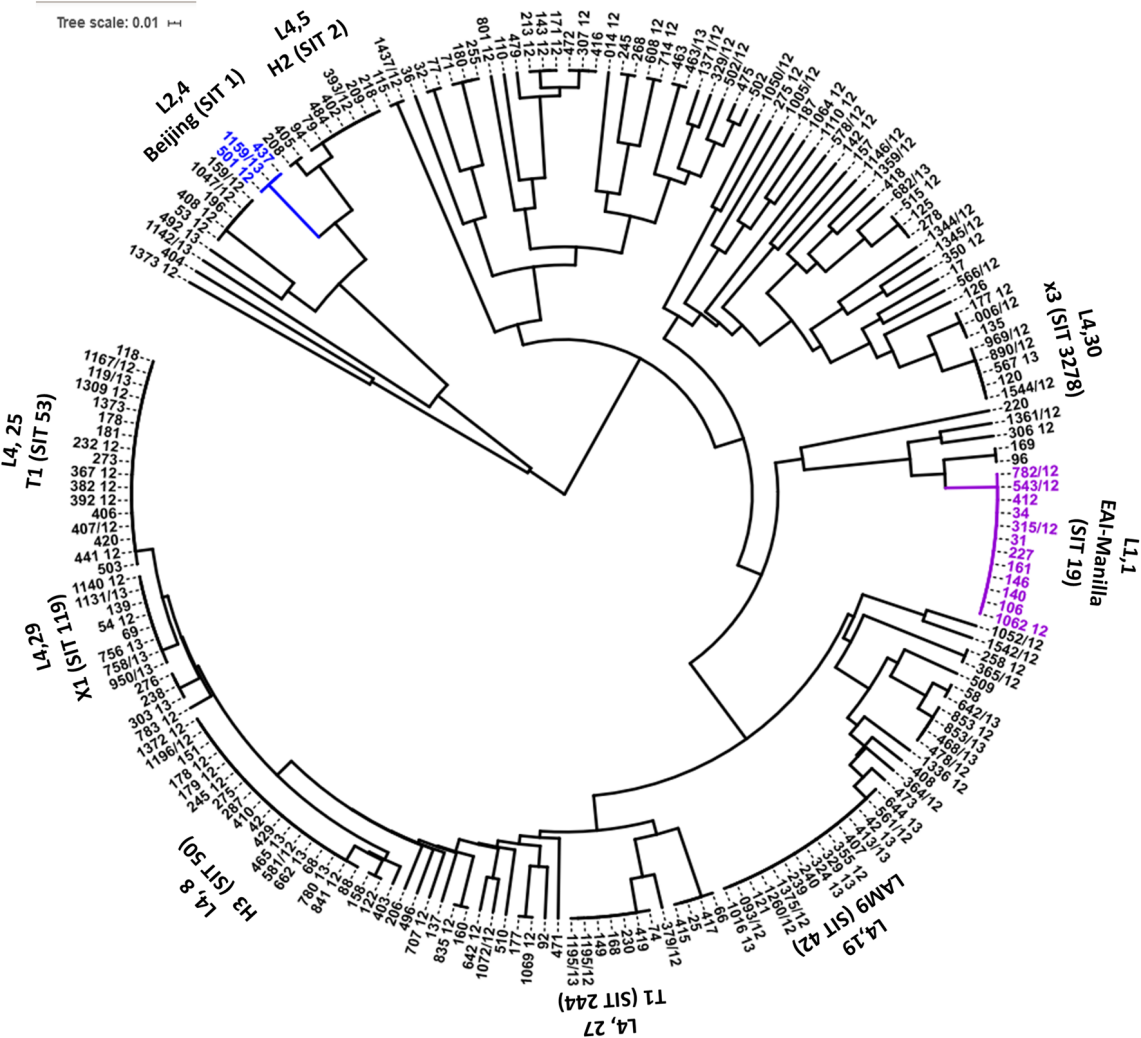
Circular tree showing the genetic relationship among 202 *M. tuberculosis *isolates based on their corresponding spoligotype typing pattern and cluster formation

The global analysis of the genotypes found shows the presence of three major lineages (Fig. 1): The first lineage was L1 (Indo-Oceanic), including eighteen isolates (9%), of which ten share the spoligotype pattern and SIT 19 (L1.2, EAI2-Manila), one had the SIT 3277 and remaining seven isolates were absent of one SIT but classified by CBN as EAI2-Manila. The second lineage found was L2 (East Asian), containing six strains (3%), three share the same spoligotype and SIT 1 (L2.2, Beijing), and remaining three isolates were classified by CBN as MANU2 or MANU3 (Table 2). The third lineage found was the predominant, L4 (Euro-American), including 176 strains (87%) (Table 2). The detailed analyses show the occurrence of five major sublineages organized as follows: Sublineage X (L4.1.1) found in 25 strains (12%) with 12 spoligotypes divided into three clades; X1, X2 and X3. Sublineage H (L4.1.2), including the largest number of isolates 61 (32%), with 21 spoligotypes, in four clades; H1, H2, H3 and H/U. Sublineage LAM (L4.3) account 37 (18%) strains, with 14 spoligotypes, placed in five clades LAM1, LAM3, LAM4, LAM9, and LAM11. Sublineage T (L4.8) include 46 isolates (23%) in 22 spoligotypes, included in the clades; T1, T2, T3, H37Rv, and T1-RUS2. By last, sublineage S (L4.4) showed the lowest frequency with four (2%) isolates including SIT 707 and three spoligotypes, followed by sublineage Tur (L4.2.2) with two isolates (1%) bearing the same spoligotype and SIT 41.

According to clustering by spoligotyping, 147 isolates (73%) were placed in 31 clusters (consisting of 2–18 strains), the clustering rate was 58% (Table 3). Sublineage H included the largest number of isolates clustered, with 51 (25%) strains placed in twelve clusters, the cluster L4-8 (H3, SIT 50), was the most abundant including eighteen strains (9%). The second most abundant sublineage with isolates clustered was T, including 30 isolates (15%) placed in four clusters, and the second most abundant cluster L4-25 (T1, SIT 53) including seventeen strains (8%). LAM was the sublineage with the third most abundant number of isolates clustered, with twenty-seven strains, placed in five clusters, including the cluster L4-19 (LAM9, SIT 42), with fifteen (7%) isolates (Table 3).

**Table 3:**
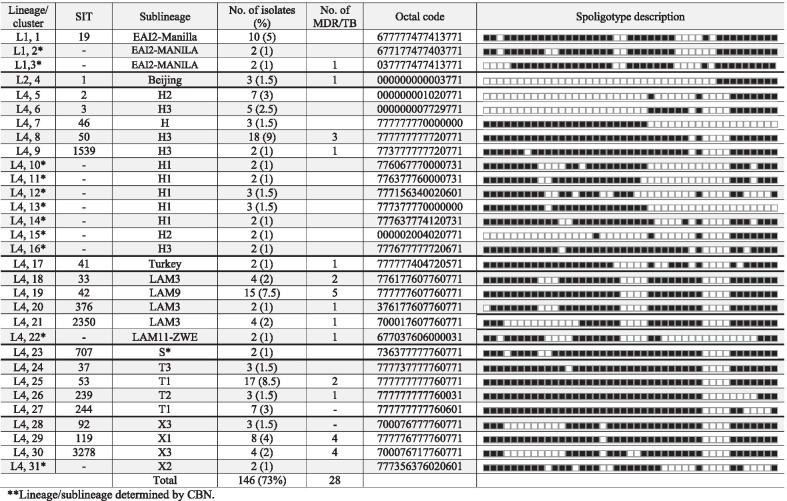
Clusters defined by spoligotyping and Tb-lineage in M. tuberculosis isolates from Veracruz city, Mexico

### MIRU-VNTR typing

Of the 202 isolates analyzed only 91 were placed in 22 clusters with 24 locus MIRU-VNTR remaining, giving a clustering rate of 34%. Meanwhile, considering the use of 15 locus MIRU-VNTR, 68 isolates were placed in 18 clusters, giving a clustering rate of 25% (Additional file 1: Table S1).

According to the HGDI values MIRU QUB26 (0.85), and QUB11b (0.84) was the most discriminatory locus, mine while the least discriminatory was MIRU02 (0.35). The HGDI at each MIRU-VNTR locus varied significantly. Sixteen loci exceeded 0.6 h, suggesting that they were highly discriminating, (2165: ETRA, 3690: Mtub39, 2531: MIRU23, 2461: ETRB, 2347: Mtub29, 1644: MIRU16, 2996: MIRU26, 3192: MIRU31, 2401: Mtub30, 802: MIRU40, 4156: QUB4156, 580: MIRU04, 960: MIRU10, 424: Mtub04, 2163b: QUB11b and 4052: QUB26). Remaining eight loci (154 MIRU02, 2059 MIRU20, 2687 MIRU24, 577 ETRC, 3171 Mtub34, 3007 MIRU27, 1955 Mtub21, and 4348 MIRU39), showed moderate discrimination (0.3 ≤ *h* ≤ 0.6).

### Genotyping characterization of the MDR-TB isolates

Of the 38 MDR-TB isolates identified, 28 (74%) were placed in 15 clusters, the rest were singletons. The four sublineages with the higher number of clusters containing TB-MDR isolates were: (1) sublineage X, with two clusters including eight MDR-TB isolates (cluster L4-29 and L4-30). (2) sublineage LAM, with seven MDR-TB isolates in two clusters (cluster L4-19 and L4-18) (Table 3). No epidemiological or clinical associations with any cluster or sublineage in the MDR-TB isolates were identified.

## Discussion

The present study provides the first description of the genetic structure of *M. tuberculosis* in Veracruz city and its metropolitan area. This region comprises a 1, 642 km^2^ surface, includes 800,000 inhabitants, is bordering the Gulf of Mexico, and is the city with the most important port activity in the East of the country. The TB prevalence is 42 per 100,000 inhabitants, besides, it concentrates most of the DR and MDR-TB cases in the state, and the country. For these reasons is considered as one of the hotspot cities for TB and MDR-TB in Mexico.

Of the 202 strains studied, 61 (30%) were resistant to at least one drug, and 38 (18%), were multi-drug resistant, also was found that 191 individuals (95%) were new cases. These data confirm the contribution of this region to the TB problem and the magnitude of the primary resistant transmission. Additional studies will be necessary to evaluate the factors that participating in this transmission.

At the phylogenetic level, the Euro-American lineage (L4) was predominant among isolates studied with 60%. This is consistent with previous studies, in which this lineage is described as highly frequent in several regions from Mexico [8, 9, 11–17], and Latin American countries [32, 33]. This success in the transmission of this lineage has been explained as a consequence of the European colonization, in the middle of the sixteenth century, with the importation of lineages and sublineages [26, 32, 34], and also in terms of its adaptation to the immune response of the host in the region [35].

Inside the Euro-American lineage, six sublineages were identified: H, LAM, T, X, TUR and S, including fifteen groups (H1, H2, H3, and H/U, LAM1, LAM3, LAM4, LAM9, T1, T2, T3, h37Rv, T1-RUS, X1, X3, TUR and S). Most of these sublineages had been described in several locations of the country, confirming their role in shaping the genetic structure of *M. tuberculosis* [8–16] in Mexico.

Lineage L1 (Indo-Oceanic) was the second most abundant phylogenetic group found, comprising 9% of the strains analyzed (18).This low frequency of occurrence is in agreement with previous reports from other settings in the country, however, it is important to mention that isolates with this lineage seems to be increased in cities with important international port activities [9, 14, 16, 18]. Specifically, Veracruz City is placed bordering the Gulf of Mexico and has one of the most important seaport activities in the country, keeping important commercial, touristic, and migration activities with countries from South America, Europe, Africa, and West Asia. Therefore, the frequency of isolates with L1, and the potential association with migratory activities should be analyzed in detail considering more resolutive tools such as WGS.

The third group found was lineage L2, East Asian, including 3% (n = 6) of isolates. Three isolates were considered as MANU, being the first description of these spoligotypes in the country. In addition, three isolates were classified as Beijing (SIT1), confirming that this lineage is widely distributed in the country [12, 14, 16, 17]. Sublineage Beijing has been documented to have higher virulence, mortality and a strong tendency to develop drug resistance, even causing up to 50% of TB cases in East-Asia [36]. The low frequency observed for this lineage in Veracruz city may be due to a poor adaptation to the local population or a recent introduction in the area. There is no doubt that the identification and characterization of this lineage will have important epidemiological implications in this city, so molecular epidemiological studies should be continued to identify transmission and infection routes.

Out of 202 isolates analyzed, 140 (69%) were included in 42 spoligotype patterns with a previously reported SIT. The most abundant were SIT 50 (H3, n = 18), followed by SIT 53 (T1, n = 17) and SIT 42 (LAM9, n = 15), all these SITs have been commonly described in several states from the North, Center, and South of Mexico, and several countries from America and Europe (Table 2). These three SITs also included the 62% of DR-TB and 50% of the MDR-TB cases identified and could be considered as important participants in the transmission of TB in the city.

The diversity of SITs observed, the low cluster index of 58% and the important number of isolates with no previous SIT description in the country, emphasize the diversity of the genetic structure of TB observed in the City of Veracruz. With no doubt, the inclusion of a greater number of isolates, and the inclusion of more powerful tools of analysis, such as WGS, will help to confirm the diversity of the genotypes found, and paths of transmission. This information will help to explain with more detail, the high prevalence of TB in this city and the potential participation of the migration or the international trade.

An important failure of this study was the no identification of associations related to socio-demographic, clinical, and lineage conditions. The only exception was the association between resistance against pyrazinamide and clustering (*p* = 0.001) (Additional file 2: Table S2). This behavior has been frequently described in previous reports with Mexican isolates and explained in terms of the fragmentation of the sample by the occurrence of an important number of sublineages [13, 17]. Undoubtedly, the number of isolates must be increased to generate sufficient data to identify the risk factors involved in the transmission of specific genotypes.

## Conclusions

In conclusion, this work provides, for the first time, the description of the genetic structure of *M. tuberculosis* strains circulating in a hotspot city of TB in Mexico by using spoligotyping and MIRU-VNTR-typing methods. Two were the major findings, the first related with the strong participation of Euro American lineage, accounted for over two-thirds of tubercle bacilli studied, confirming the main role of this lineage in shaping the genetic structure of TB and second was related with the high diversity of sublineages and genotypes observed in circulation.

## Supplementary Information

**Additional file 1: Table S1.** Clonal complexes defined by MIRU-VNTR of *M. tuberculosis *strains from Veracruz city, México.

**Additional file 2: Table S2.** Sociodemographic characteristics of individuals bearing an isolate clustered and no clustered from the city of Veracruz, México.

## Data Availability

The datasets used and/or analyzed during the current study available from the corresponding author under request.

## Abbreviations

TB: Tuberculosis
WHO: World Health Organization
DR-TB: Drug resistant tuberculosis
MDR-TB: Multidrug resistant tuberculosis
RR-TB: Rifampicin resistant tuberculosis
MIRU-VNTR: Mycobacterial interspersed repetitive units-variable number of tandem repeats
AFB: Acid Fast Bacilli
S: Streptomycin
H: Isoniazid
R: Rifampin
E: Ethambutol
Z: Pyrazinamide
BCG: Bacillus Calmette-Guerin
HGDI: Hunter-Gaston Diversity Index
UPGMA: Unweighted pair group method with arithmetic mean
SPSS: Statistical Package for the Social Sciences
SIT: Spoligotype international type
