# Supplementary material for: Genetic diversity and drug susceptibility of Mycobacterium tuberculosis in a city with a high prevalence of drug resistant tuberculosis from Southeast of Mexico
Source: BMC Infect Dis. 2021 Nov 30;21:1202. doi: 10.1186/s12879-021-06904-z (PMC8630842; doi:10.1186/s12879-021-06904-z)
Supplement: Supplementary file 1 — Additional file 1: Table S1. Clonal complexes defined by MIRU-VNTR of M. tuberculosis strains from Veracruz city, México. [file 12879_2021_6904_MOESM1_ESM.docx]

**Table S1. Clonal complexes defined by MIRU-VNTR of *M. tuberculosis* strains from Veracruz city, México**

| **CC**  **24 loci** | **MIRU-VNTR pattern** | **No. of isolates** | **MIRU-VNTR pattern**  **15 loci** | **No. of isolates** | **Lineage (SIT), Cluster** |
| --- | --- | --- | --- | --- | --- |
| 1 | 223236333732425154343633 | 14 | 232363373454363 | 13 | H3 (50), cluster18 |
| 2 | 243242432335236254344942 | 12 | 432424333254494 | 10 | T1 (53), cluster 25 |
| 3 | 254214232224116154443733 | 9 | 542842322154373 | 8 | LAM9 (SIT 42 ), cluster 19 |
| 4 | 253244432234225154333743 | 6 | 532444333254394  532444323252354 | 2  2 | X1 (119), cluster 29  X1(119), cluster 29 |
| 5 | 324234523621433164433442 | 5 | 242343262463344 | 4 | H2 (2), cluster 5 |
| 6 | 224215422535435154341733 | 4 | 242154243454173 | 3 | T1 (244),cluster 27 |
| 7 | 264235333535425153333772 | 4 | 342353353453373  642353353453377 | 2  2 | H3 (3), cluster 6  H3 (3), cluster 6 |
| 8 | 263244342335535254344644 | 4 | 632443433554485 | 3 | X3 (3278), cluster 30 |
| 9 | 224525492353176223352623 | 4 | 145254985123262 | 5 | EAI2-Manila (19), cluster 1 |
| 10 | 233236433535425164443233 | 3 | - | - | T3 (37) Cluster 24 |
| 11 | 233245432334235153443941 | 3 | 332454333254394 | 2 | H1 (46), cluster 7 |
| 12 | 214525492853176224332624 | 3 | - | - | EAI2-Manila (19), cluster 1 |
| 13 | 214223322424225146333523 | 2 | - | - | LAM3 (2350), cluster 21 |
| 14 | 234213422434125152445412 | 2 | - | - | T2 (239), cluster 26 |
| 15 | 473256432234435245543545 | 2 | 732364323444354 | 2 | H1*, cluster 13 |
| 16 | 223236332732323163343832 | 2 | 232363373364383 | 2 | H1*, cluster 14 |
| 17 | 223234232532323163323433 | 2 | 236342353362343 | 2 | EAI2-MANILA, cluster 2 |
| 18 | 324334312254225255553732 | 2 | 243343125255373 | 2 | H1*, cluster 10 |
| 19 | 334344212354225255553732 | 2 | 343442135255373 | 2 | H1*, cluster 11 |
| 20 | 223244433532433255343404 | 2 | - | - | H3*, cluster 16 |
| 21 | 244234333334436254423643 | 2 | - | - | Turkey (41), cluster 17 |
| 22 | 223233332334325344333533 | 2 | 235333333343353 | 2 | LAM11-ZWE*, cluster 22 |
|  | **Total** | **91** |  | **68** |  |
