# Supplementary material for: Genetic diversity and drug susceptibility of Mycobacterium tuberculosis in a city with a high prevalence of drug resistant tuberculosis from Southeast of Mexico
Source: BMC Infect Dis. 2021 Nov 30;21:1202. doi: 10.1186/s12879-021-06904-z (PMC8630842; doi:10.1186/s12879-021-06904-z)
Supplement: Supplementary file 2 — Additional file 2: Table S2. Sociodemographic characteristics of individuals bearing an isolate clustered and no clustered from the city of Veracruz, México. [file 12879_2021_6904_MOESM2_ESM.docx]

|  | |  |  |  |  |  |  |  |
| --- | --- | --- | --- | --- | --- | --- | --- | --- |
| **Table S2. Sociodemographic characteristics of individuals bearing an isolate clustered and no clustered from the city of Veracruz, México** | | | | | | | | |
| **Variable** | | | **Total**  ***n*= 202 (%)** | **Clustered**  ***n*= 147 (%)** | **No clustered**  ***n*=55 (%)** | **Fisher exact test** | ***p*** | ***P***  **(Yates´s correction)** |
| Sex | Male  Female | | 138 (68.3%)  64 (31.7%) | 99 (69.2%)  44 (30.8%) | 39 (66.1%)  20 (33.9%) | 0.740 | 0.664 | 0.635 |
| Age (years) | Mean ±SD  ≥ 36  < 35 | | 41.6 (±16.2)  132 (66.3%)  67 (33.7%) | 40.8 (±16.1)  90 (63.8%)  51 (36.2%) | 43.5 (±16.8)  42 (72.4%)  16 (27.6%) | 0.322 | 0.244 | 0.179 |
| Alcoholism | Yes  No | | 17 (8.4%)  185 (91.6%) | 13 (9.1%)  130 (90.9%) | 4 (6.8%)  55 (93,2%) | 0.782 | 0.591 | 0.941 |
| T2DM comorbidity | Yes  No | | 42 (20.8%)  160 (79.2%) | 27 (18.9%)  116 (81.1%) | 15 (25.4%)  44 (74.6%) | 0.341 | 0.297 | 0.678 |
| HIV comorbidity | Yes  No | | 2 (1.0%)  200 (99.0%) | 2 (1.4%)  141 (98.6%) | 0 (0.0%)  59 (100%) | 1.000 | 0.361 | - |
| H | Yes  No | | 52 (25.7%)  150 (74.3%) | 42 (29.4%)  101 (70.6%) | 10 (16.9%)  49 (83.1%) | 0.066 | 0.078 | 0.336 |
| R | Yes  No | | 41 (20.3%)  161 (79.7%) | 32 (22.4%)  111 (77.6%) | 9 (15.3%)  50 (84.7%) | 0.252 | 0.336 | 0.581 |
| P | Yes  No | | 25 (12.4%)  177 (87.6%) | 22 (15.4%)  121 (84.6%) | 3 (5.1%)  56 (94.9%) | *0.058* | *0.043* | *0.0041* |
| E | Yes  No | | 19 (9.4%)  183 (90.6%) | 13 (9.1%)  130 (90.9%) | 6 (10.2%)  53 (89.8%) | 0.795 | 0.811 | 0.859 |
| DR | Yes  No | | 60 (29.7%)  142 (70.3%) | 48 (33.6%)  95 (66.4%) | 12 (20.3%)  47 (79.7%) | 0.065 | 0.061 | 0.157 |
| MDR | Yes  No | | 39 (19.3%)  163 (80.7%) | 30 (21.0%)  113 (79.0%) | 9 (15.3%)  50 (84.7%) | 0.435 | 0.349 | 0.342 |

*resistance with significant difference, *p=*<0.001
